# Estimating *B*_1_^+^ in the breast at 7 T using a generic template

**DOI:** 10.1002/nbm.3911

**Published:** 2018-03-23

**Authors:** Michael J. van Rijssel, Josien P. W. Pluim, Peter R. Luijten, Kenneth G. A. Gilhuijs, Alexander J. E. Raaijmakers, Dennis W. J. Klomp

**Affiliations:** ^1^ Center for Image Sciences UMC Utrecht Utrecht The Netherlands

**Keywords:** 7 T, *B*_1_^+^ mapping, breast, flip‐angle correction, RF field, *T*_1_ mapping

## Abstract

Dynamic contrast‐enhanced MRI is the workhorse of breast MRI, where the diagnosis of lesions is largely based on the enhancement curve shape. However, this curve shape is biased by RF transmit (B_1_^+^) field inhomogeneities. B_1_^+^ field information is required in order to correct these. The use of a generic, coil‐specific B_1_^+^ template is proposed and tested.

Finite‐difference time‐domain simulations for B_1_^+^ were performed for healthy female volunteers with a wide range of breast anatomies. A generic B_1_^+^ template was constructed by averaging simulations based on four volunteers. Three‐dimensional B_1_^+^ maps were acquired in 15 other volunteers. Root mean square error (RMSE) metrics were calculated between individual simulations and the template, and between individual measurements and the template. The agreement between the proposed template approach and a B_1_^+^ mapping method was compared against the agreement between acquisition and reacquisition using the same mapping protocol.

RMSE values (% of nominal flip angle) comparing individual simulations with the template were in the range 2.00‐4.01%, with mean 2.68%. RMSE values comparing individual measurements with the template were in the range8.1‐16%, with mean 11.7%. The agreement between the proposed template approach and a B_1_^+^ mapping method was only slightly worse than the agreement between two consecutive acquisitions using the same mapping protocol in one volunteer: the range of agreement increased from ±16% of the nominal angle for repeated measurement to ±22% for the B_1_^+^ template.

With local RF transmit coils, intersubject differences in B_1_^+^ fields of the breast are comparable to the accuracy of B_1_^+^ mapping methods, even at 7 T. Consequently, a single generic B_1_^+^ template suits subjects over a wide range of breast anatomies, eliminating the need for a time‐consuming B_1_^+^ mapping protocol.

**Abbreviations used**
*B*_1_^+^: RF transmit field
DCE‐MRI: dynamic contrast‐enhanced MRI
DESPOT1: driven‐equilibrium single‐pulse observation of *T*_1_
DREAM: dual refocusing echo acquisition mode
RMSE: root mean square error
SAR: specific absorption rate
SD: standard deviation
*T*_1_NR: *T*_1_‐to‐noise ratio.

## 1 INTRODUCTION

Dynamic contrast‐enhanced MRI (DCE‐MRI) is the workhorse of clinical breast MRI examinations. Since its introduction in the 1980s, it has become a standard in breast MRI examinations due to its robustness and ability to detect tumor malignancy.^1^, ^2^ This ability is based on the differences in dynamics of contrast agent uptake between tumors and healthy parenchymal tissue, leading to characteristic enhancement curve shapes. Automated analysis of these curves enabled the introduction of computer aided diagnosis methods into clinical practice, and pharmacokinetic models have been proposed to quantify the exchange of contrast agents between the inflowing blood and surrounding tissue.^3^, ^4^, ^5^

Taking breast DCE‐MRI to higher field strengths such as 7 T is currently being investigated, showing potential for earlier and more accurate diagnosis.^6^ The higher signal‐to‐noise ratio that is available at higher field strengths enables higher spatial resolution. A higher resolution not only permits detection of smaller lesions, but also improves assessment of the heterogeneity of contrast uptake, such as rim enhancement, which is associated with worse survival in triple negative breast cancers.^7^ It was shown that using the available signal‐to‐noise ratio to achieve a higher temporal resolution at 7 T is also feasible in a clinical setting.^8^

A fast wash‐out rate is a typical indication of a malignant tumor, while a stable curve or continued wash‐in often reflects benign lesions. However, the curve shape can be compromised by RF transmit (*B*_1_^+^) field variations, potentially shifting the curve of a tumor that should have caused a wash‐out shape into a more stable curve. This can be conceptually understood by considering the fact that at different *B*_1_^+^ levels there are different amounts of *T*_1_ saturation, and the effects of a change in *T*_1_ (due to contrast administration) will differ. If we define *B*_1_^+^ induced image intensity bias as $\frac{\text{measured intensity}}{\text{true intensity}\;\left(B_{1}^{+}=100\%\right)}$, then for fast RF spoiled gradient echo sequences bias=sinB1+θnom1−e−TRT1cosθnomsinθnom1−e−TRT1cosB1+θnom. Observe that this bias depends not only on *B*_1_^+^, but also on *T*_1_, which in DCE‐MRI is not constant in time, and the image intensity bias will change over the dynamic series. Generally, for any *B*_1_^+^ below 100%, the bias increases with increasing *T*_1_; consequently, when the *T*_1_ of tumor tissue drops due to contrast injection, the DCE curve's wash‐in is reduced due to the counteracting effect of the intensity bias. The opposite effect occurs when due to contrast wash‐out the tumor's *T*_1_ rises again, leading to a compromised curve, shifted to appear more stable than the true curve.

Correction for this *B*_1_^+^ effect is possible, using *B*_1_^+^ field maps and an estimate of *T*_1_ before contrast injection.^9^ It has been shown that applying *B*_1_^+^ correction at 3 T has a significant effect on the results of quantitative analysis and serves to reduce differences in quantitative parameter estimations between the right and left breasts.^10^ Recent work shows that, even at 1.5 T, refraining from *B*_1_^+^ field corrections leads to a 50% estimation error in tumor *T*_1_ and consequently a 41% estimation error in pharmacokinetic parameters.^11^ At 7 T, the *B*_1_^+^ field variations manifest themselves on a smaller spatial scale, such that variations within a single breast become significant. Therefore, when applying DCE‐MRI at 7 T, corrections using *B*_1_^+^ field maps are imperative.

*B*_1_^+^ field variations are much more significant at higher field strengths due to the reduced wavelength of the RF field. At 7 T, the proton excitation frequency is 300 MHz, leading to an RF wavelength of around 15 cm inside the body (assuming a relative permittivity around 60). The breasts, however, contain high amounts of fat, which has a relative permittivity that is an order of magnitude lower than that of most other tissues. This leads to a longer RF wavelength inside the breasts, approximately 40 cm assuming a relative permittivity around 10. Such a wavelength is usually larger than the size of the imaged anatomy. In this case, the *B*_1_^+^ field distribution within the breast will depend mainly on the local transmit setup used, and hardly at all on the individual anatomy. Therefore, we hypothesize that one generic, coil‐specific *B*_1_^+^ template will suit a wide range of subjects in the case of breast examinations with local transmit coils. We set out to test our hypothesis at 7 T, where local transmit is a commonly used strategy to overcome RF inhomogeneity issues.

The advantages of using a generic *B*_1_^+^ template in a clinical setting are twofold. It eliminates the need to acquire a *B*_1_^+^ field map, saving scan time. Furthermore, *B*_1_^+^ mapping techniques are known to be prone to noise and many are only reliable within a certain range.^12^, ^13^ Since the generic template is based on (partly simulated) data of multiple subjects, it is essentially noise free and reliable in the full range of *B*_1_^+^ inhomogeneities present. Though other techniques to estimate *B*_1_^+^ in breast without acquiring field maps exist, these methods often rely on fat as a reference tissue (with a fixed *T*_1_) in order to estimate *B*_1_^+^ in the parenchyma.^14^, ^15^ Such methods may not be suitable for fat‐suppressed sequences and rely on extrapolation of a fitted field distribution outside fatty regions. The template method presented is not hampered by these limitations, since *B*_1_^+^ distributions can be deducted regardless of the sequence used. This method is limited only by the availability of a *B*_1_^+^ template of the coil design used.

The present work aims to explore the feasibility of using a generic *B*_1_^+^ template by investigating the inter‐subject differences in *B*_1_^+^ inhomogeneity. The work comprises both simulated field maps and measured ones, in order to compare template performance with the accuracy of *B*_1_^+^ mapping.

## 2 METHODS

In order to test our hypothesis that one generic, coil‐specific *B*_1_^+^ template will suit a wide range of subjects when performing breast MRI with local transmit coils, a number of experiments were performed. First, RF simulations from previous work were used to create the template (Section 2.1). Next, *B*_1_^+^ and *T*_1_ mapping was performed on 15 new volunteers (Section 2.2) and their breast volume and composition were estimated (Section 2.3). Section 2.4 describes *Q* measurements of the coil that we conducted to investigate the influence of breast anatomy on coil loading. The acquired *B*_1_^+^ maps were used to compare against the predictions made using the constructed template (Section 2.5). Finally, we tested the ability of the *B*_1_^+^ template predictions to correct *T*_1_ maps for *B*_1_^+^ inhomogeneity corruption against *B*_1_^+^ map corrected *T*_1_ measurements (Section 2.6), using methodology that is common in quantitative DCE‐MRI.

All experiments conducted were in accordance with the guidelines of the local ethical committee and, prior to the examination, written informed consent was obtained from all volunteers.

### 2.1 Simulations and template construction

**Figure 1:**
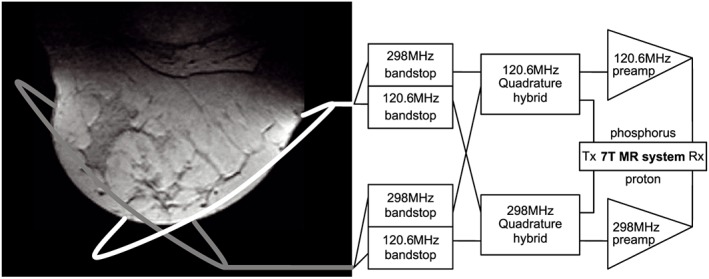
Schematic overview of the dual‐channel unilateral breast coil for the detection of ^31^P and ^1^H MR signals. The locations of the two elements are illustrated by two ellipsoids on a transverse MR image of the human breast. These elements are interfaced to the transmit (Tx) and receive (Rx) line of the 7 T MR system using bandstop filters, quadrature hybrids and preamplifiers as illustrated on the right. Reproduced from Reference 16

The coil setup used in this work was a quadrature setup as illustrated in Figure 1 and presented in earlier work by Klomp et al.^16^ Their work also demonstrates the high efficiency of this coil and its usefulness in imaging and spectroscopy applications for 7 T breast MRI.

**Figure 2:**
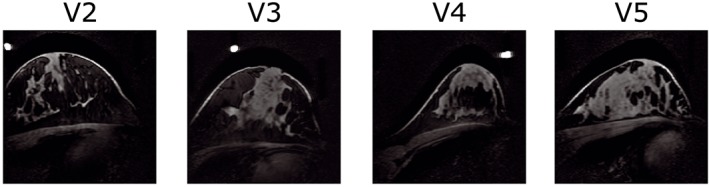
Fat‐suppressed T_1_w scans of the four included simulation volunteers (sagittal view). Bright dots mark the locations of vitamin tablets used to determine the position of the coil by van der Velden et al.^17^

Finite‐difference time‐domain simulations of *B*_1_^+^ and *B*_1_^‐^ distributions in five healthy female volunteers (V1‐V5), presented in previous work, were used to investigate inter‐subject differences in *B*_1_^+^ distribution at 7 T when using this local transmit coil setup.^17^ In short, *B*_1_ field distributions were calculated from personalized breast segmentations obtained from T1w Dixon scans fused with Virtual Family model Ella, and a 3D model of the relevant MR equipment.^18^, ^19^ Segmentations of glandular tissue, adipose tissue and skin were assigned their corresponding dielectric permittivity and conductivity values.^20^ Finite‐difference time‐domain simulations were conducted for 201 000 time steps of 3 × 10^−12^ s (one Larmor period) with a mesh of 2 × 2 × 2 mm^3^, assuming perfectly absorbing boundary layers. Convergence was assessed by visual inspection. Coil losses were not considered, since these have no impact on *B*_1_^+^ distributions. Vitamin tablets were attached to the coil elements using adhesive tape, in order to identify their position in the T1w scans. Though a limited number of volunteers were used to conduct the simulations, volunteers were selected to represent a reasonably wide range in breast anatomies, as can be appreciated from their T1w gradient echo scans in Figure 2 and anatomical characteristics in Table 1. Due to missing data, the simulation for V1 had to be excluded.

**Table 1:** Inclusion table reporting the breast volume and volumetric glandular percentage for all simulation volunteers (V2‐V5)

Number	Breast volume (cm^3^)	Gland percentage
V2	756	17%
V3	777	29%
V4	285	33%
V5	474	44%
**Mean**	**573**	**31%**
**SD**	**205**	**9%**
**Min.**	**285**	**17%**
**Max.**	**777**	**44%**

In order to directly compare the simulated *B*_1_^+^ and *B*_1_^‐^ fields, which did not share a common coordinate system, all simulated field distributions were aligned using multi‐resolution intensity‐based rigid registration in elastix.^21^ For this purpose, a mutual information similarity metric, third‐order *B*‐spline interpolation and an adaptive stochastic gradient descent optimizer were used. This allows transmit coil alignment, since these coils are present in the simulations as hyperintense fields closest to the coil conductors. Differences in RF amplification settings between volunteers were overcome by normalizing all simulations relative to the simulation of V5 (arbitrarily chosen). In order to do so, the following intensity scaling factor was applied to the simulated field distribution for volunteers V2‐V5:

$$\text{scale}\left(i\right)=\text{median}\left\{\forall \vec{r}:\frac{{\text{simulation}}_{5}\left(\vec{r}\right)}{{\text{simulation}}_{i}\left(\vec{r}\right)}\right\}$$

where *i* is the volunteer number and $\vec{r}=\left(x,y,z\right)$ the position in the simulation. Note that the median was preferred over the mean to ensure robustness against outliers.

Subsequently, the average of the rescaled simulation distributions of volunteer V2‐V5 was taken, we denote these the *B*_1_^+^ and *B*_1_^‐^ templates. Differences between volunteers were assessed per volunteer by comparing each individual simulation result with the *B*_1_^+^ template. The comparison was quantified using mean difference, standard deviation (SD) of difference and root mean square error (RMSE) metrics. All metrics were calculated over all voxels within the breast region of interest, which was determined previously.^17^ The *B*_1_^‐^ template was created to facilitate *B*_1_^+^ template scaling using information obtained from the power optimization phase.

### 2.2 Scanning protocol

In order to prospectively compare the accuracy of the template approach with *B*_1_^+^ mapping, a validation set of 15 additional healthy female volunteers (S1‐S15), mean age 39 years (range 24‐62, all ages are reported in Table 2), were scanned in the prone position using the same unilateral breast coil setup on a 7 T whole‐body MR system (Achieva; Philips, Cleveland, OH, USA).^16^ A 3D *B*_1_^+^ map was acquired using the dual refocusing echo acquisition mode (DREAM) technique with the following parameters: pulse repetition time 4.0 ms, stimulated echo time 1.49 ms, free induction decay echo time 1.97 ms, 2.5 mm isotropic resolution, preparation angle 55°, imaging angle 25° and turbo field echo factor 32.^12^ For three volunteers, S13‐S15, the DREAM *B*_1_^+^ acquisition was repeated, to compare the accuracy of the proposed template method with the variation between repeated measures. 3D *T*_1_‐weighted gradient echo images were acquired at four flip angles (2°, 4°, 13° and 27°) using Dixon water‐fat separation with the following parameters: in‐phase echo time 1.97 ms, out‐phase echo time 4.4 ms, repetition time 6.0 ms and isotropic resolution 1.5 mm.^19^ Both scans were planned according to a fast survey scan; measurements obtained during this scan's power optimization phase were logged and later used in template scaling. Scan parameters for both sequences are summarized in Table 3. The *B*_1_^+^ map and variable flip angle images allowed the calculation of *T*_1_ maps using the driven‐equilibrium single‐pulse observation of *T*_1_ relaxation (DESPOT1) technique^22^; see the *T*_1_ mapping section for more details and the rationale behind the choice of angles.

**Table 2:** Inclusion table reporting the age, breast volume, volumetric glandular percentage and ratio *Q*_unloaded_/*Q*_loaded_ for all validation volunteers (S1‐S15)

Number	Age (years)	Breast volume (cm^3^)	Gland percentage	*Q*_unloaded_/*Q*_loaded_
S1	24	495	28%	—
S2	26	479	28%	3.2
S3	24	638	64%	5.2
S4	25	382	26%	3.7
S5	30	309	25%	3.7
S6	33	213	38%	3.9
S7	57	184	17%	3.0
S8	62	570	10%	—
S9	45	1032	7.2%	—
S10	55	235	24%	2.8
S11	53	928	7.5%	4.5
S12	40	707	41%	—
S13	28	129	87%	—
S14	24	351	81%	—
S15	28	494	35%	—
**Mean**	**36.9**	**476**	**35%**	**3.7**
**SD**	**13.4**	**256**	**25%**	**0.74**
**Min.**	**24**	**129**	**7.2%**	**2.8**
**Max.**	**62**	**1032**	**87%**	**5.2**

**Table 3:** Summary of scan parameters per sequence

Sequence	*T*_R_ (ms)	*T*_E_ (ms)	Resolution (mm^3^)	Flip angle (°)	Other parameters
DREAM *B*_1_^+^ mapping	4	SE: 1.49FID: 1.97	2.5 × 2.5 × 2.5	Preparation: 55Imaging: 25	TFE acceleration factor: 32
Dual‐echo gradient echo (4×)	6	IP: 1.97OP: 4.4	1.5 × 1.5 × 1.5	2/4/13/27	Dixon reconstruction: water & fat images
Survey (3D, fast RF spoiled gradient echo)	6	1.25	3 × 3 × 10	10	Reconstructed to 2 × 2 × 5 mm^3^

### 2.3 Estimating breast volume and composition

Estimates of breast volume and composition were calculated for all volunteers. Since the pectoral muscle was not visible for all volunteers due to the limited range where the coil transmits and receives sufficient signal, estimates of breast volume were obtained using the method described by Katariya et al.^23^ on transversal maximum intensity projections. Though this method is rather simplistic and potentially imprecise, it has been shown to be highly reproducible and correlated with mastectomy excision volume, and allows for comparison with published population data.^24^, ^25^ The Dixon water and fat reconstructions were used to estimate volumetric gland percentage for each volunteer.

### 2.4 *Q*‐factor measurements

In order to check individual differences of coil loading, all volunteers (S1‐S15) were asked to return on a different day for additional *Q*‐factor measurements. Out of 15, nine volunteers were able to participate but in one volunteer the measurement failed, leading to eight useable data points. Measurements were made using a purpose‐built coil that contained a replica of the innermost element of the coil that was used in the MR experiments. The mechanics of the setup were identical to those used in the scanner. The *Q* factor (defined as central resonance frequency over bandwidth) was determined using a network analyzer. Volunteers were asked to lie down in the prone position on the setup as they did in the scanner, positioning the arms on their back. Values for *Q* both with and without loading were recorded for each volunteer; the ratio *Q*_unloaded_/*Q*_loaded_ was calculated as a measure for coil loading. The ratios were plotted against breast volume and volumetric glandular percentage for each volunteer, and a trend line was calculated using analytical ordinary least squares estimation.

### 2.5 Comparing *B*_1_^+^ template and measured maps

Rigid registration was applied to the *B*_1_^+^ template to facilitate direct comparison with the measured *B*_1_^+^ map for every volunteer. The map was masked before registration to exclude regions where a *B*_1_^+^ reconstruction was not available. The template was masked by thresholding to exclude values corresponding to flip angles below 20% and above 100% of the nominal flip angle. All values higher than 100% are very close to or in coil elements; the bottom cut‐off of 20% was empirically chosen to avoid registration of the edge of the map to the edge of the template. In the resulting binary image, a 3D connected components algorithm using a 6‐connected neighborhood was used to find connected regions. The largest connected component was selected as the mask. Multi‐resolution intensity‐based rigid registration was applied in elastix, using a mutual information similarity metric, *B*‐spline interpolation and an adaptive stochastic gradient descent optimizer.

Subsequently, the *B*_1_^+^ values of the registered template were intensity scaled using information from the scanner's power optimization phase. During this phase, a global *B*_1_^+^ level (PO *B*_1_^+^) for the sample is measured. Using scanner log data from the power optimization phase for all volunteers, a calibration line was determined between this global PO *B*_1_^+^ and an average *B*_1_^+^ value determined from the registered template, scaled to match the measured *B*_1_^+^ map (i.e. the best possible template scale for every volunteer). Taking into account the global nature of the PO *B*_1_^+^, the average *B*_1_^+^ value was weighted with both *B*_1_^+^ and *B*_1_^−^: $\text{weighted}\left|B_{1}^{+}\right|=\frac{\sum_{\forall r\in M}\left(\left(B_{1}^{-}\left(r\right)B_{1}^{+}\left(r\right)\right)B_{1}^{+}\left(r\right)\right)}{\sum_{\forall r\in M}\left(B_{1}^{-}\left(r\right)B_{1}^{+}\left(r\right)\right)}$, where *M* is a mask created by thresholding the survey scan using Otsu's method.^26^ The calibration line obtained in this fashion was subsequently used to scale each registered *B*_1_^+^ template, independently of its measured *B*_1_^+^ map.

The map and the registered template were compared on individual bases through calculation of the RMSE, mean error and SD of the error per volunteer and a total mean absolute error for the validation set. Additionally, a Bland‐Altman density plot was created, showing the agreement between the measured *B*_1_^+^ map and the registered and scaled template for all 15 volunteers (S1‐S15). As suggested by Bland and Altman, the same kind of plot was created for two repeated DREAM‐based *B*_1_^+^ mapping measurements to study repeatability.^27^ This allowed for comparison of the limits of agreement between the template method and the DREAM method with the degree of variation between repeated *B*_1_^+^ mapping procedures for volunteers S13‐S15.

### 2.6 *T*_1_ mapping

As is commonly done in quantitative DCE‐MRI, we used *B*_1_^+^ maps to correct for the effects of *B*_1_^+^ inhomogeneities using a variable flip‐angle *T*_1_ mapping method.^10^, ^11^, ^22^, ^28^, ^29^ This method uses several *T*_1_‐weighted gradient echo scans at different flip angles to estimate the *T*_1_ value at every recorded voxel by performing a fit of the signal equation, which is a function of the applied flip angle. Since this is a voxel‐wise method, *B*_1_^+^ correction can be easily applied by fitting the function while using the actual flip angle as the independent variable, i.e. the nominal angle multiplied by the value in the *B*_1_^+^ map for that voxel.

**Figure 3:**
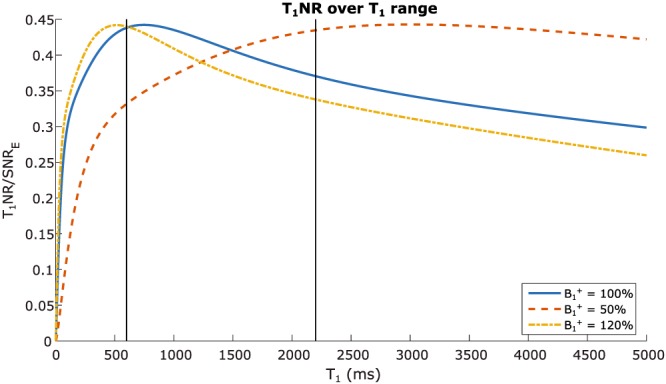
T_1_NR of the variable‐flip‐angle acquisition scheme, when using the DESPOT1 analysis method. Profiles for *T*_1_‐to‐noise are plotted for three levels of *B*_1_^+^; vertical lines indicate the *T*_1_ values for glandular tissue (2200 ms) and fat (600 ms)

The flip‐angle combination was chosen by taking into consideration the notions put forth by Deoni et al.,^30^ ensuring an accurate *T*_1_ measurement over the wide *B*_1_^+^ range (50‐120% of the nominal angle) and the wide *T*_1_ range (600‐2200 ms) present in the breast. To determine the best flip‐angle combination, all combinations of four integer angles in the range 1‐90° were tested and the *T*_1_‐to‐noise ratio (*T*_1_NR) was calculated for every combination of angles with *T*_1_ set to either 600 or 2200 ms and *B*_1_^+^ set to either 50% or 120% of the nominal angle. The sum over all four combinations of *B*_1_^+^ and *T*_1_ for *T*_1_NR determined the suitability of every combination of angles. Figure 3 shows the *T*_1_NR using the selected flip angle combination (2°, 4°, 13° and 27°) over a wide range of *T*_1_ values for three levels of *B*_1_^+^.

*T*_1_ maps were calculated from the data using the DESPOT1 method.^22^ In the fitting procedure, the independent variable was either the nominal angle, the angle as measured by the DREAM sequence or the angle as predicted by the template. This leads to *T*_1_ maps that are not corrected for *B*_1_^+^, corrected by the DREAM *B*_1_^+^ data or corrected by the generic *B*_1_^+^ template respectively. The SD of the *T*_1_ estimate was calculated in every voxel, following the methodology described in Reference 30. An estimate of the noise level was obtained by taking the SD of the image intensity in anatomy‐free regions of the gradient echo images. Finally, all voxels for which the SD in the *T*_1_ estimate was larger than 100 ms were (empirically) considered unreliable and excluded (the average exclusion percentage was 10.3% of all voxels inside the region where the DREAM *B*_1_^+^ map was defined).

The obtained *T*_1_ maps were analyzed by comparing the measurements corrected using the measured map versus using the template. The *T*_1_ estimates were compared on individual bases through calculation of the RMSE, mean error and SD of the error.

## 3 RESULTS

Table 1 shows the breast volume and volumetric glandular percentage of all simulation volunteers; Table 2 shows the same for all validation volunteers. In the simulation set, breast volume ranged from 285 to 777 cm^3^ and glandular percentage from 17 to 44%; in the validation set, breast volume ranged from 129 to 1032 cm^3^ and glandular percentage from 7 to 87%.

**Figure 4:**
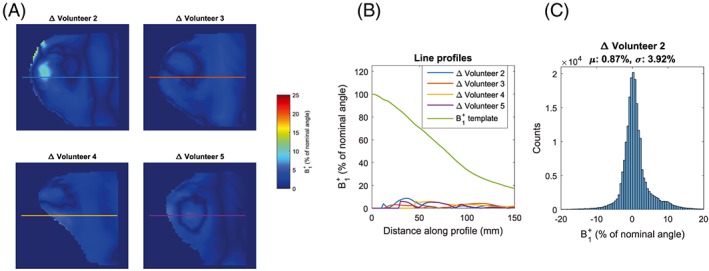
A, Absolute difference between mean *B*_1_^+^ (template) and individual simulations. B, Line profiles corresponding to same‐colored lines in A. C, Histogram of difference between template and simulation of Volunteer 2 (which showed least agreement)

The difference between the constructed generic template and every individual volunteer's simulation is shown in Figure 4. Panel B shows that the differences between individuals are small particularly compared with the large dynamic range in *B*_1_^+^ in each individual. As Table 4 shows, the mean RMSE between the generic template and individual simulations was 2.68% of the nominal angle, while in the worst agreeing volunteer (V2) this was 4.01%.

**Table 4:** Comparison between individual *B*_1_^+^ simulations and the generic template for all simulation volunteers (V2‐V5)

Number	Mean error (% of nom. angle)	SD of error (% ona)	RMSE (% ona)
V2	0.87	3.92	4.01
V3	0.80	2.24	2.38
V4	−1.42	1.85	2.34
V5	0.35	1.97	2.00
**Mean**	**0.15**	**2.50**	**2.68**
**SD**	**0.93**	**0.83**	**0.78**
**Min.**	**−1.42**	**1.85**	**2.00**
**Max.**	**0.87**	**3.92**	**4.01**

% ona, percentage of nominal angle.

Measurements for *Q*_unloaded_/*Q*_loaded_ ranged from 2.8 to 5.2. Measurements per volunteer are reported in Table 2.

**Figure 5:**
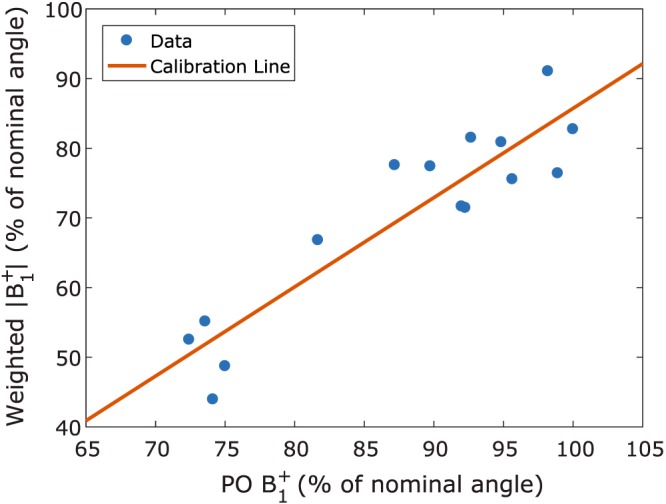
Calibration line for *B*_1_^+^ template scaling. The global *B*_1_^+^ measured during the scanner's power optimization phase is regressed against a weighted average of the *B*_1_^+^ template, scaled to match the measured *B*_1_^+^ map. Adjusted *R* ^2^ of the fit is 0.825

**Figure 6:**
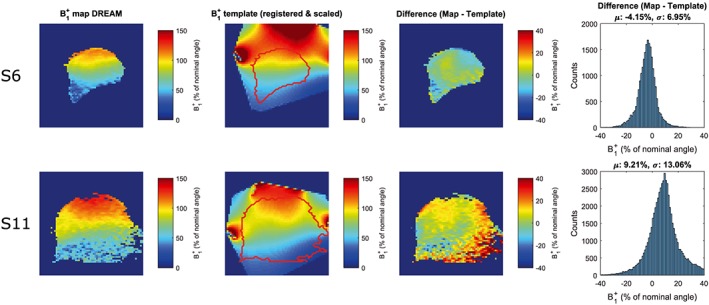
For volunteer S6 (top row) and volunteer S11 (bottom row), from left to right: *B*_1_^+^ map measured with DREAM technique; *B*_1_^+^ template registered and scaled to measured map (the red line indicates the breast outline as in the first panel); difference between template and map; histogram of difference between template and map

**Figure 7:**
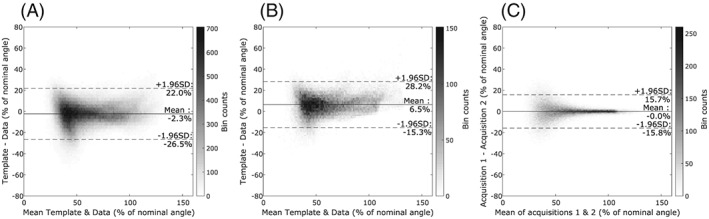
A, Bland‐Altman analysis of the registered templates and measured maps for all volunteers (S1‐S15). The data is visualized using a density histogram; the scale bar on the right indicates the amount of counts in each bin. B, Bland‐Altman analysis of a subset of the data displayed in A; only volunteers S13‐S15 are shown. C, Bland‐Altman analysis of repeatedly measured *B*_1_^+^ maps of volunteers S13‐S15

The calibration line used in power‐optimization‐based scaling of the template is shown in Figure 5. The calibration line fit had an adjusted *R* ^2^ of 0.825. The (registered and scaled) generic template and the measured *B*_1_^+^ map are similar, as can be appreciated visually from Figure 6. It shows both the best matching case (S6) and the worst matching case (S11), based on the RMSE. Table 5 shows statistics for all volunteers (S1‐S15). The mean RMSE between the generic template and individual prospective measurements was 11.7% of the nominal angle; the total mean absolute error was 5.37%. The Bland‐Altman analysis of all volunteers in Figure 7A shows that the measured maps and generic template agree less in regions with low *B*_1_^+^ than areas with high *B*_1_^+^. Figure 7B shows the same analysis for a subset; only data from volunteers S13‐S15 has been included. Figure 7C shows a Bland‐Altman analysis of repeated DREAM *B*_1_^+^ mapping for the same volunteers (S13‐S15); note that the limits of agreement are 12% wider in B than in C.

**Table 5:** Comparison between individual *B*_1_^+^ measurements and the generic template for all validation volunteers (S1‐S15)

	Mean error (% of nom. angle)	SD of error (% ona)	RMSE (% ona)
S1	−1.9	11	11
S2	7.9	9.4	12
S3	−0.93	12	12
S4	4.9	8.3	9.6
S5	3.9	7.5	8.4
S6	−4.2	7.0	8.1
S7	−6.3	8.4	10
S8	3.0	10	10
S9	5.6	12	14
S10	2.2	9.0	9.3
S11	9.2	13	16
S12	8.8	12	15
S13	−10	11	15
S14	−5.5	11	13
S15	−6.1	11	12
**Mean**	**0.710**	**10.2**	**11.7**
**SD**	**5.96**	**1.85**	**2.34**
**Min.**	**−10**	**7.0**	**8.1**
**Max.**	**9.2**	**13**	**16**

% ona, percentage of nominal angle.

**Figure 8:**
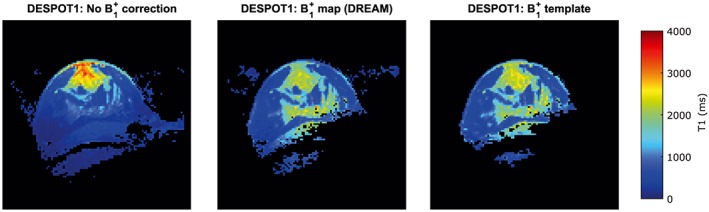
For volunteer S1, from left to right: *T*_1_ map using DESPOT1 and no *B*_1_^+^ correction; *T*_1_ map using DESPOT1 and *B*_1_^+^ map measured with DREAM; *T*_1_ map using DESPOT1 and *B*_1_^+^ template registered and scaled to measured map

*T*_1_ estimates calculated with DESPOT1 and either map‐based or template‐based *B*_1_^+^ information are close, as can be appreciated visually in Figure 8 for Volunteer S1. Table 6 shows a quantitative analysis for all volunteers (S1‐S15); the mean RMSE was 318 ms.

**Table 6:** Comparison between the DREAM‐corrected and template‐corrected measurements of *T*_1_ (ms) for all validation volunteers (S1‐S15)

	Mean error (ms)	SD of error (ms)	RMSE (ms)
S1	−54	171	180
S2	213	255	332
S3	−80	231	244
S4	160	215	268
S5	146	295	329
S6	−144	201	248
S7	−232	261	349
S8	−47	225	230
S9	−66	184	196
S10	−23	286	287
S11	−110	165	199
S12	−181	353	397
S13	−586	359	687
S14	300	400	500
S15	187	272	331
**Mean**	**−34**	**258**	**318**
**SD**	**213**	**68**	**128**
**Min.**	**−586**	**165**	**180**
**Max.**	**300**	**400**	**687**

## 4 DISCUSSION

Our results from the simulations show that differences in *B*_1_^+^ distributions in the breast between volunteers covering a wide anatomical range are generally very small, only of the order of 2‐3%. This amount of variation is of the same order of magnitude as the accuracy of popular *B*_1_^+^ mapping methods.^31^ This is a strong indication that, in breast MRI with local RF transmit coils at 7 T or lower, resorting to field mapping for every subject is unnecessary.

The width of the anatomical range of the validation set, quantified in Table 2, is substantial, in terms of both breast volume and glandular percentage. Comparing our distribution of breast volumes to that of 225 healthy women in Reference 24 shows that we have captured a wide range, except for the high end of the spectrum, since the maximum included volume is only1 SD away from the reported average; sadly, our coil setup and non‐wide‐bore MR system are incapable of accommodating women with higher breast volumes. The included range of breast densities is also broad when compared with a representative group of 531 consecutively included patients receiving breast MRI; the largest reported volumetric glandular percentage is 50% in Reference 32. Note that Gubern‐Mérida et al. showed that volumetric estimations of breast density based on MRI tend to underestimate BI‐RADS (Breast Imaging Reporting and Data System) density scores, and in their study of 132 women from a high‐risk group with age characteristics comparable to those in our study none of the subjects had a percentage higher than 60%.^33^ The width of the simulation set is inevitably smaller, due to the small number of four volunteers included. Yet it captures a reasonably wide range (at least 1 SD from the average for both breast density and volume) and, arguably, our results obtained with only the limited simulation set only make the case for a template‐based *B*_1_^+^ estimation stronger.

Our results from the measured *B*_1_^+^ maps show that a generic template can accommodate volunteers over a wide range of breast anatomies. The Bland‐Altman analysis in Figure 7A makes it clear that the measured maps are interchangeable with the registered and scaled generic template, within the range between the limits of agreement (−26.5% to +22.0% of the nominal angle). In other words, if one is satisfied with an error between the two methods up to approximately 20% of the nominal angle, the methods may be exchanged. Note that the range of agreement is considerably narrower (and thus better) if one were to exclude regions where *B*_1_^+^ is low, where the measured maps are unreliable.^12^ In those regions, the generic template might actually be at an advantage, since it does not suffer from such a limitation and is noise‐free in nature. To investigate whether the observed limits of agreement between the proposed template method and the DREAM method are acceptable, Figures 7B and 6C show the results for three volunteers (S13‐S15) of an identical Bland‐Altman analysis between measured data and proposed template (6B) and between measured data and repeated measurement (6C). The range between the limits of agreement is slightly larger for the template than for repeated measurements: where repeated measurements had an error of up to 15% of the nominal angle for volunteers S13‐S15, this range increased by approximately 6% using a *B*_1_^+^ template. The bias that can be observed in Figure 7B is mainly due to the use of the calibration line for template scaling, and this bias will differ for each volunteer. Note from Table 5 that volunteers S13‐S15 all have quite large mean errors, which explains the high mean offset (bias) in Figure 7B; for most subjects, this bias will be smaller.

It is clear from Figure 8 that the *B*_1_^+^ corrected DESPOT1‐based *T*_1_ maps are substantially more homogeneous in both lipids and glandular tissue, irrespective of whether the *B*_1_^+^ information is from a map or the template. When comparing the analyses in Table 5 and Table 6 it is clear that the mean errors in *B*_1_^+^ propagate into mean errors in *T*_1_ estimates. In all cases but one, the SD of the error distribution is bigger than the mean error, which means that the two measurements of *T*_1_ do not significantly differ.

A limitation of this study is the fact that we have to rely on RF simulations to be able to construct the template. Several studies, however, have shown that these kinds of simulation are able to accurately predict *B*_1_^+^ distributions and show high agreement between measured and simulated field maps.^34^, ^35^, ^36^, ^37^ The fact that all simulations were performed using a single body model with different breast models may further impact the validity of our simulations, though since local transmit coils were used the effect will be limited to an increase or decrease of the total efficiency. If this effect is present, it will be corrected by the intensity scaling of the registered template (Section 2.4). The results of the *Q* measurements also contribute to this conclusion. While they show that tissue load is dominant (*Q*_unloaded_/*Q*_loaded_ was around 4 for all volunteers), they also show that, even with breast volume changes by up to a factor of 5, load variations were all within 30% of *Q*_unloaded_/*Q*_loaded_ = 4. This means that the biggest tissue load is caused by the rest of the body and that the *Q* variance over breast anatomies is limited, analogous to our results in *B*_1_^+^. Probably the RF eddy currents that occur in the rest of the body predominantly have a local *B*_1_^+^ effect that is either of insufficient strength to affect the *B*_1_^+^ in the breast, or hardly differs from the effects observed in the Virtual Family model (Ella).

Though this paper only demonstrates the use of a template for a unilateral breast coil, we believe that this can be extended to bilateral cases. Hardware developments in high‐field MRI tend to go towards parallel multi‐transmit systems, where amplitude and phase of all coil elements can be steered individually. In such setups, regarding each breast independently in terms of *B*_1_^+^ is a reasonable assumption.

It is of note that demonstrating agreement between measured and simulated *B*_1_^+^ distributions is often used to validate predictions in specific absorption rate (SAR). While our work shows that *B*_1_^+^ distributions in the breast are very similar from person to person, the same does not necessarily hold for SAR. In fact, in recent work by Alon et al., it was demonstrated that *B*_1_^+^ distributions tend to be correlated over samples, but the same did not hold for SAR.^38^ Therefore, they conclude that using *B*_1_^+^ distributions to validate SAR predictions should be done with caution. The present work serves as further proof of the statement that *B*_1_^+^ distributions tend to have high correlations between subjects; this is the very phenomenon we exploit when constructing and using a *B*_1_^+^ template.

Implementation of the generic template approach in a clinical setting requires knowledge of both the position of the transmit coil in the image and the amount of template scaling that is needed for each subject. In many setups, the position of the coil is fixed on the bed, eliminating the positioning problem altogether. We have solved the scaling issue by using readily available information from the power optimization phase and the survey scan, information that will be present in any clinical protocol. This strategy brings a dependence of the template's performance on the goodness of fit of the calibration line of Figure 5: a large variation from the calibration line causes a large mean error (bias) in the resulting template‐based *B*_1_^+^ distribution. As reported in Table 5, the SD of the mean error was 5.96% of the nominal angle; in the ideal situation where the scaling is calculated directly from a measured *B*_1_^+^ map, this SD reduces to 1.78% of the nominal angle. The main reason for the reduced performance (and the goodness of fit of the calibration line) might be that the *B*_1_^+^ measured during the power optimization procedure is non‐localized. Therefore, it was assumed that the measured *B*_1_^+^ level during the power optimization was a weighted average over the entire imaged region that contained tissue. The differences in mean error between volunteers may be further reduced when a localized power optimization method is employed for template scaling.^39^

In conclusion, simulations show that inter‐subject differences in *B*_1_^+^ fields of the breast at 7 T are comparable to the accuracy of popular *B*_1_^+^ mapping methods reported in literature. Consequently, we have shown that, at the cost of a small loss in accuracy (the range of agreement increased from ±16% of the nominal angle for repeated measurement to ±22% for the *B*_1_^+^ template), using a generic *B*_1_^+^ template to account for substantial RF transmit inhomogeneity in *T*_1_ mapping may be feasible across a wide range of volunteers.
